# Distributed Fiber-Optic Strain Sensing of an Innovative Reinforced Concrete Beam–Column Connection

**DOI:** 10.3390/s22103957

**Published:** 2022-05-23

**Authors:** Shenghan Zhang, Han Liu, Esam Darwish, Khalid M. Mosalam, Matthew J. DeJong

**Affiliations:** 1 Department of Civil and Environmental Engineering, The Hong Kong University of Science and Technology, Clear Water Bay, Kowloon, Hong Kong, China; ceshenghan@ust.hk; 2 Department of Civil and Environmental Engineering, University of California, Berkeley, CA 94720, USA; han_liu@berkeley.edu (H.L.); mosalam@berkeley.edu (K.M.M.); 3 Structural Engineering Department, Tanta University, Tanta 31527, Egypt; essam.darwish@f-eng.tanta.edu.eg

**Keywords:** damage detection, damage assessment, distributed fiber-optic sensing, reinforced concrete structure, beam–column connection

## Abstract

Distributed fiber-optic sensing (DFOS) technologies have been used for decades to detect damage in infrastructure. One recent DFOS technology, Optical Frequency Domain Reflectometry (OFDR), has attracted attention from the structural engineering community because its high spatial resolution and refined accuracy could enable new monitoring possibilities and new insight regarding the behavior of reinforced concrete (RC) structures. The current research project explores the ability and potential of OFDR to measure distributed strain in RC structures through laboratory tests on an innovative beam–column connection, in which a partial slot joint was introduced between the beam and the column to control damage. In the test specimen, fiber-optic cables were embedded in both the steel reinforcement and concrete. The specimen was tested under quasi-static cyclic loading with increasing displacement demand at the structural laboratory of the Pacific Earthquake Engineering Research (PEER) Center of UC Berkeley. Different types of fiber-optic cables were embedded both in the concrete and the rebar. The influence of the cable coating and cable position are discussed. The DFOS results are compared with traditional measurements (DIC and LVDT). The high resolution of DFOS at small deformations provides new insights regarding the mechanical behavior of the slotted RC beam–column connection, including direct measurement of beam curvature, rebar deformation, and slot opening and closing. A major contribution of this work is the quantification of the performance and limitations of the DFOS system under large cyclic strains. Performance is quantified in terms of non-valid points (which occur in large strains when the DFOS analyzer does not return a strain value), maximum strain that can be reliably measured, crack width that causes cable rupture, and the effect of the cable coating on the measurements. Structural damage indices are also proposed based on the DFOS results. These damage indices correlate reasonably well with the maximum sustained drift, indicating the potential of using DFOS for RC structural damage assessment. The experimental data set is made publicly available.

## 1. Introduction

After a disastrous event (e.g., an earthquake), cities are burdened with the need to quickly evaluate infrastructure damage. For modern cities, the indirect cost from business interruption often exceeds the cost from structural damage. Current evaluation procedures for structural damage still largely rely on the judgment of civil engineers, which is expensive and time-consuming. Meanwhile, in recent years, various types of infrastructure-sensing technologies have been developed, such as terrestrial laser scanning [1], unmanned aerial vehicle imagery [2], and distributed fiber-optic sensing (DFOS) [3,4]. DFOS, in particular, has already been successfully applied in civil infrastructure to measure distributed temperature and deformation and to detect damage [5]. However, many previous applications employ BOTDR or FBG technology, which is limited in its spatial resolution. The recent development of DFOS technologies (e.g., pulse pre-pump Brillouin optical time domain analysis (PPP-BOTDA) [6]) bring finer spatial resolution and higher accuracy and frequency, which provides the possibility to more precisely detect local damage and to transform from damage detection to damage quantification.

Damage quantification for reinforced concrete (RC) structures require a deep understanding of the mechanical behavior of the structural component. One emerging DFOS technology, Optical Frequency Domain Reflectometry (OFDR), is especially suitable for this task due to its unprecedented spatial resolution (around 1 mm) and high accuracy (around 1 μϵ). Since its appearance a few years ago, it has been used in structural engineering laboratories around the world [7,8,9,10,11]. For a detailed review, please refer to Bado and Casas [12]. Despite the recent advances, the application of DFOS in RC structures is still in its initial stage. Notably, only a few studies (e.g., Refs. [13,14]) investigated the influence of the position of the fiber-optic cable in RC components (e.g., adjacent to rebar or placed at certain distance from rebar). In addition, there is a limited number of detailed investigations (e.g., [15,16]) on the influence of fiber coating (fiber-optic cable jacket) on the sensing results. This paper contributes to addressing the aforementioned challenges by testing an innovative beam–column joint instrumented with DFOS.

A beam–column connection was selected for investigation due to its significant influence on the strength and overall stability of RC-framed structures, as observed in past earthquakes (e.g., the 1995 Kobe earthquake [17] and 2009 L’Aquila earthquake [18]). A review of the code requirements for beam–column connections can be found in [19]. The behavior of beam–column connections under cyclic loading has been extensively studied (e.g., Refs. [20,21,22]). These studies and many others usefully quantify the global structural performance of these connections, but measurement of detailed mechanical behavior was limited to discrete strain measurements. Further, innovative beam–column connection detailing to limit or control damage is still the subject of ongoing research, and understanding damage propagation in these RC beam–column connections is limited by the constraint of conventional measuring instruments (e.g., strain gauges or Linear Variable Differential Transformers (LVDTs)) [8].

The beam–column connection design adopted in this study is a partial depth slot (i.e., gap) introduced between the beam and the column to control damage. The slotted beam connection system experiences minimal beam elongation and achieves a non-tearing action under seismic loading [23,24]. The specimen was tested at the structural laboratory of the Pacific Earthquake Engineering Research (PEER) Center of UC Berkeley under quasi-static cyclic loading. Traditional measurements, including Digital Image Correlation (DIC), strain gauges, and LVDTs, were used to measure the performance of the reinforcing steel bars and the concrete. In addition, DFOS was implemented using the ODiSI 6000 Series sensing platform (Luna Innovations Inc). Four optical fibers with three different coatings were embedded in different locations of the specimen to investigate the structural behavior of the RC joint.

This paper starts with introducing the test set-up and fiber layout (Section 2). The DFOS results at three representative drift levels are then presented and discussed, including a comparison with the DIC and LVDT results (Section 3). In Section 4, several damage indices are proposed based on the DFOS results at both the maximum and residual drift levels. The correlations between the proposed damage indices and the maximum or residual drifts sustained by the RC joint are further evaluated.

## 2. Test Set-Up and Fiber Layout

**Figure 1:**
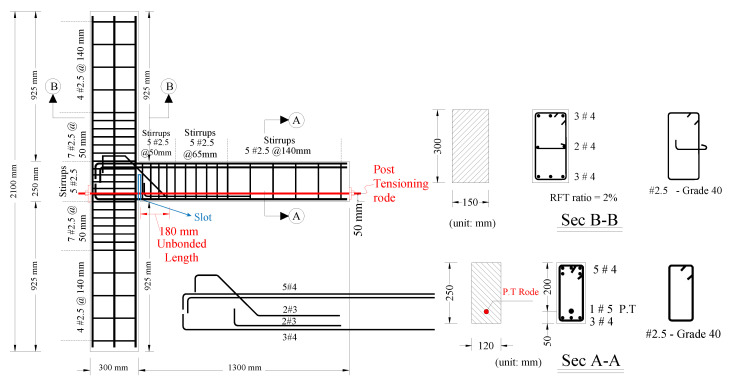
Details of slotted beam–column connection with post-tensioning.

The dimensions and reinforcement details of the tested specimen are shown in Figure 1. In addition to the slotted joint, which is a half-inch-wide gap that extends over the bottom 3/4 depth of the beam (highlighted in blue in Figure 1), the connection also features 180 mm of unbonded rebar on the beam side of the slot (highlighted in Figure 1) to enable a reduced level of strain in the rebar. This innovative design aims to reduce the plastic hinge zone damage, limit the beam elongation, and enhance the low cyclic fatigue life of the beam longitudinal bottom rebars at the unbonded length. Additionally, near the slot, there is a 3/4-inch (19.05 mm) in diameter post-tensioning (PT) rod placed at 50 mm from the bottom face of the beam (Sec A-A in Figure 1) to simulate the compression in real beams under earthquake action [25]. For more details on the slotted connection concept, see [26].

**Figure 2:**
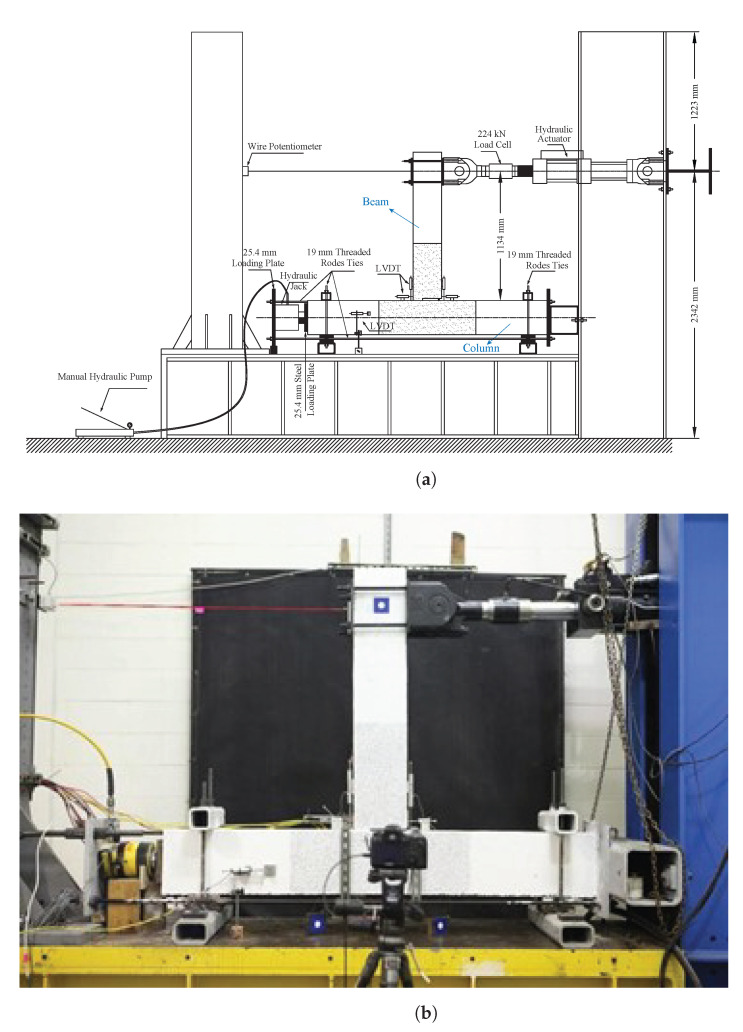
Test set-up used for the tests at PEER, UC Berkeley. (**a**) Illustrative sketch; (**b**) Photo: front side.

Figure 2 presents the test set-up. For the ease of testing, the specimen was rotated 90 degrees so the beam was vertical. The column (horizontal member in Figure 2) is loaded axially with a constant compression force of 60 kN. The cyclic loading is applied through the actuator at the top of the beam. The beam is also sustaining compression from the PT rod (post-tensioned to 65 kN of compression force before the test).

**Figure 3:**
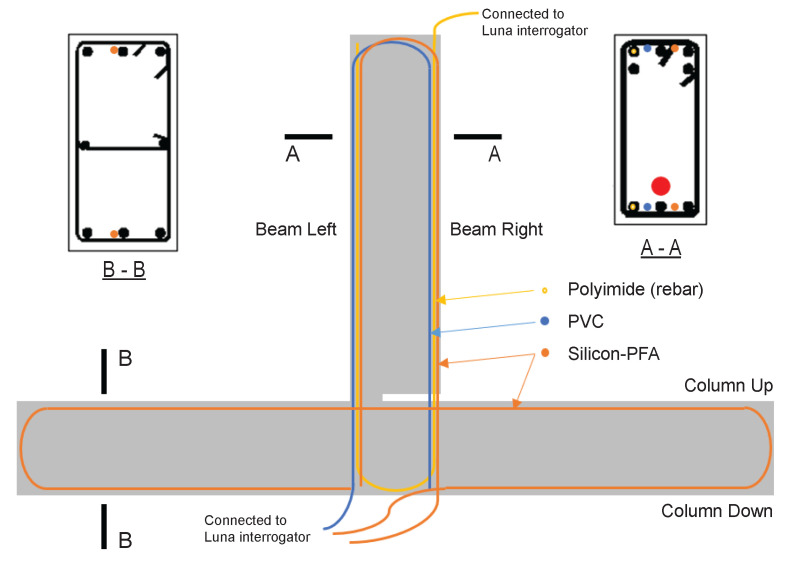
Fiber-optic cable layout in the specimen.

The layout of the optical cables is presented in Figure 3. In the beam, there are three different kinds of fiber-optic cables. Two cables are embedded in the concrete. They are fixed on the stirrups using zip ties halfway between the longitudinal reinforcing bars. One additional cable is epoxied in a cut groove in the longitudinal rebar (yellow cable in cross section A-A in Figure 3). Please refer to Zhang et al. [14] for the photos of the fiber-optic cables, as well as the process of embedding fiber-optic cable in the rebar. The two fiber-optic cables embedded in concrete are one cable from TLC Tight Buffer SM ClearCuve ZBL with PVC coating (“PVC” in Figure 3, represented by the blue color) and another cable from OFS Fitel, LLC with a silicon inner coating and a PFA outer coating (“Silicon-PFA” in Figure 3, represented by the orange color). For the cable embedded in the rebar, a polyimide-coated fiber (supplied by Luna Innovations) was used (Figure 3). In the column, only Silicon-PFA was used and was placed adjacent to the longitudinal reinforcing bar (cross section B-B in Figure 3). For all the fiber-optic cables, the spatial resolution was set to be 1.3 mm with a sampling rate of 6.25 Hz.

## 3. Fiber-Optic Sensing Results and Comparison with Digital Image Correlation

In this section, the DFOS results at the following representative drift levels are presented and discussed: ±0.14%, ±0.95%, and ±1.76%. For comparison, the DIC results at corresponding drift levels are also presented, which are obtained through the free software Optecal. While this section discusses three representative drift levels, the DFOS results at all drift levels are organized in a Matlab app which is publicly shared.

### 3.1. Drift Level = ±0.14%

**Figure 4:**
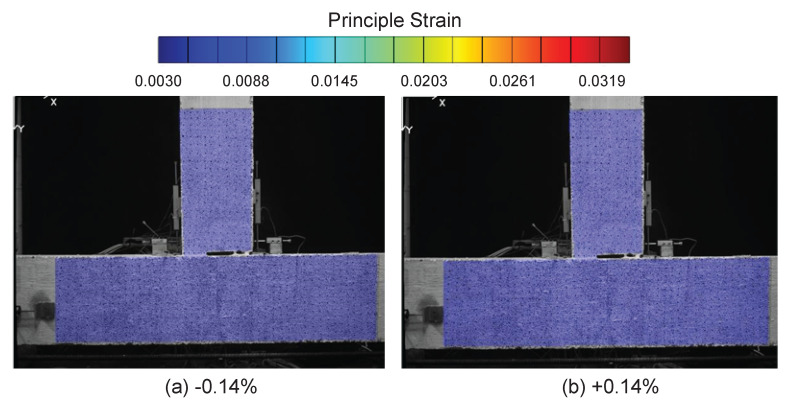
DIC results at a drift level of ±0.14%.

**Figure 5:**
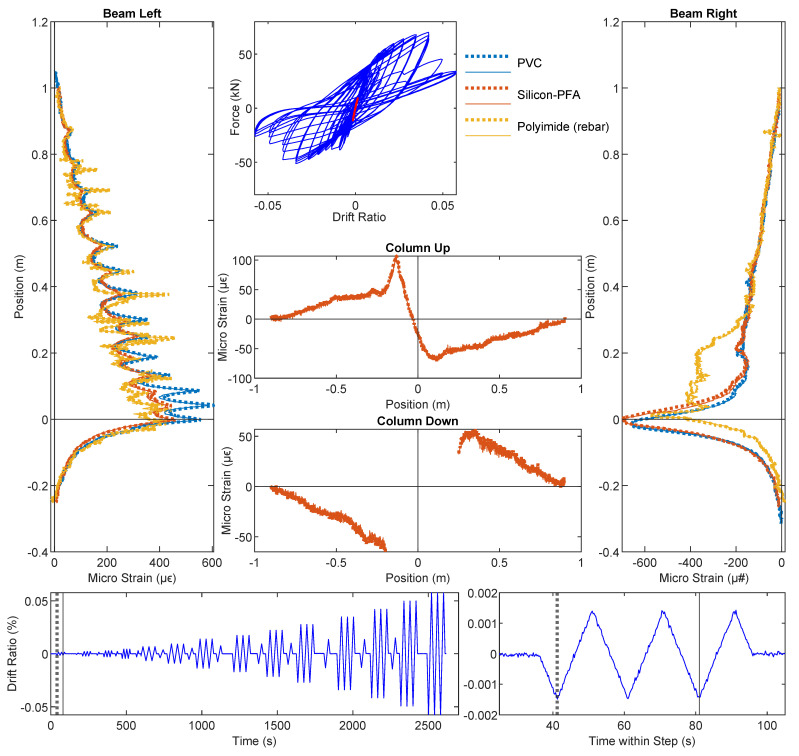
DFOS results at a drift level of −0.14%.

**Figure 6:**
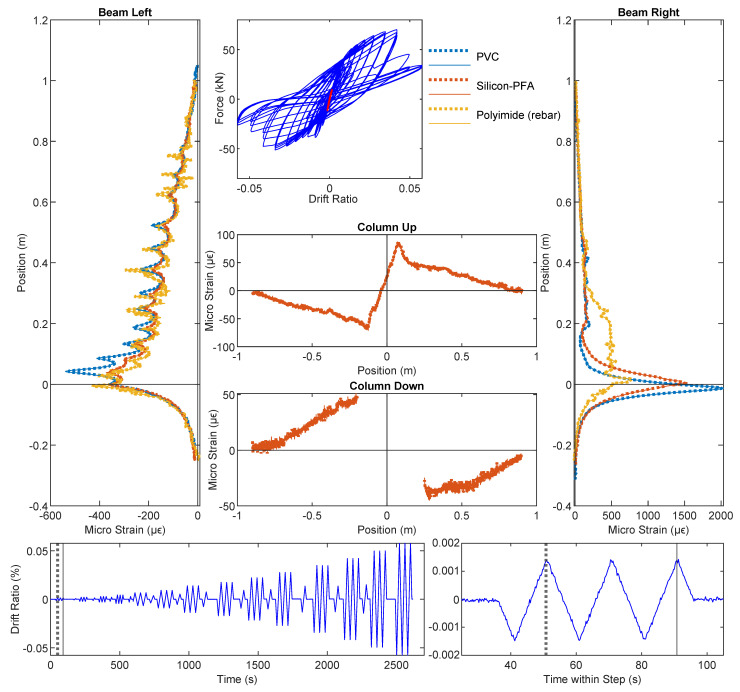
DFOS results at drift level of 0.14%.

At the drift level ± 0.14%, no visible damage or strain information can be obtained from DIC (Figure 4). However, the deformation profile along the beam and column can be clearly observed from DFOS at this drift level. The DFOS results are presented in Figure 5 and Figure 6, in which the subfigures Beam Left and Beam Right present the DFOS results at the left and right sides of the vertical beam (see Figure 3), respectively, while subfigures Column Up and Column Down show the DFOS results at the up and down sides of the horizontal column, respectively. The three colors indicate different fiber-optic cables embedded in the specimen (Figure 3).

To investigate the influence of cyclic loading on the DFOS measurements, two time instants were distinguished for the same drift level (i.e., the first time and the third time the drift reached the maximum or minimum value for a specified drift level). The two different time instants are indicated by vertical lines in the bottom subfigures in Figure 5 and Figure 6, in which the dotted thick line indicates the first time the drift reached the target level, while the solid thin line indicates the third time. The same line type was adopted to distinguish the DFOS results at these two time instants in subfigures Beam Left and Beam Right as well as Column Up and Column Down (Figure 5 and Figure 6). At this load level, the DFOS results for the first and third cycles coincided for all of the fibers. This reveals that there was no further damage under repeated loading for the current drift level of ±0.14%, and for the current drift level, all three optical cables gave reliable readings under cyclic loading.

From top to bottom along the beam, the gradual increase in strain in the optical fibers indicates increasing curvature along the beam caused by increasing moment. The asymmetric behavior of the beam is clear when comparing the maximum tension under loading in different directions (Beam Left in Figure 6 with positive drift and Beam Right in Figure 5 with negative drift): the maximum tension was higher when the slotted side (right side in Figure 3) underwent tension. More specifically, at the same drift level, the maximum tensile strain on the right side was ~2000 micro strain (Figure 6), while the maximum tensile strain was only ~500 micro strain on the left side (Figure 5), caused by the concentration of deformation near the slot.

In addition to the gradual change in strain along the beam (Figure 5 and Figure 6), local strain peaks, the spacing of which corresponded to the stirrup spacing, were clearly evident, especially along the left side of the beam. While the DFOS reading for the fiber embedded in the rebar (“Polyimide (rebar)” in Figure 5 and Figure 6) directly revealed the strain in the rebar, the measured strain from the fiber-optic cables embedded in the concrete (i.e., PVC and Silicon-PFA) resulted from the superposition of concrete strain and strain caused by cracks. In addition, the strain measurements were also influenced by deformation of the fiber coating and potential bond slip between the fiber-optic cable and the concrete [16]. Some of the larger local peaks in the concrete (Figure 6) were likely caused by micro-cracks that developed at the stirrup locations.

For the optical cable embedded in the rebar, additional noise was observed (Figure 5 and Figure 6). Given that recent experiments exhibited smoother results [14], this local noise was likely caused by the variation of the groove in the manufacturing process. It is also worth noting that the strain readings on the left side of the beam were similar for all three optical cables, while the strain distributions along the right side of the beam were different. On the right side of the beam, the rebar reading was nearly uniform near the slot because of the 180-mm unbonded length of the rebar (Figure 1). For the fibers embedded in concrete (PVC and Silicon-PFA), the influence of the cable coating is evident in Figure 5 when the slot opened. The PVC cable exhibited a slightly larger strain than the Silicon-PFA cable with the same slot opening, which indicates a higher sensitivity [14].

### 3.2. Drift Level = ±0.95%

**Figure 7:**
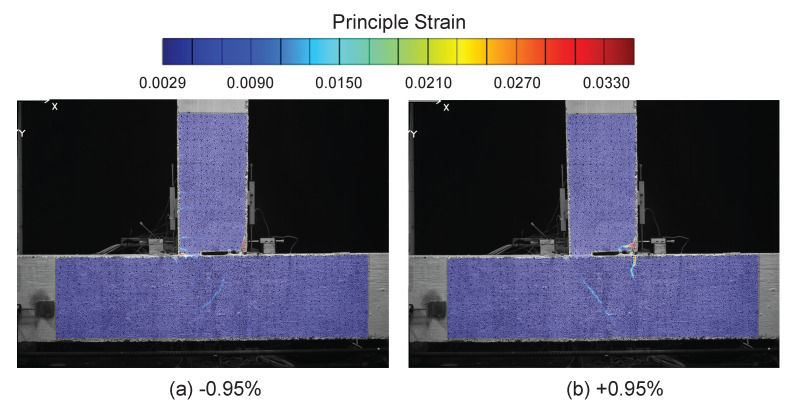
DIC results at a drift level of ±0.95%.

**Figure 8:**
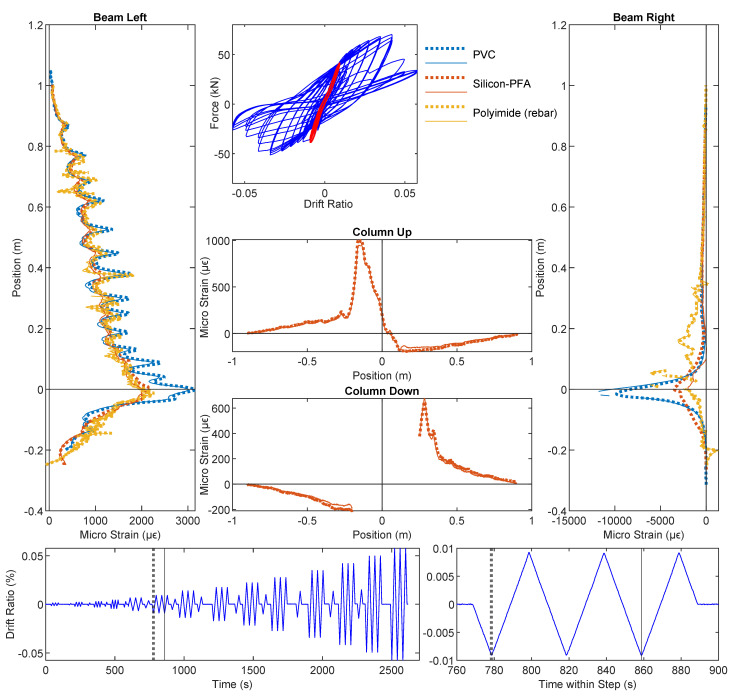
DFOS results at a drift level of −0.95%.

**Figure 9:**
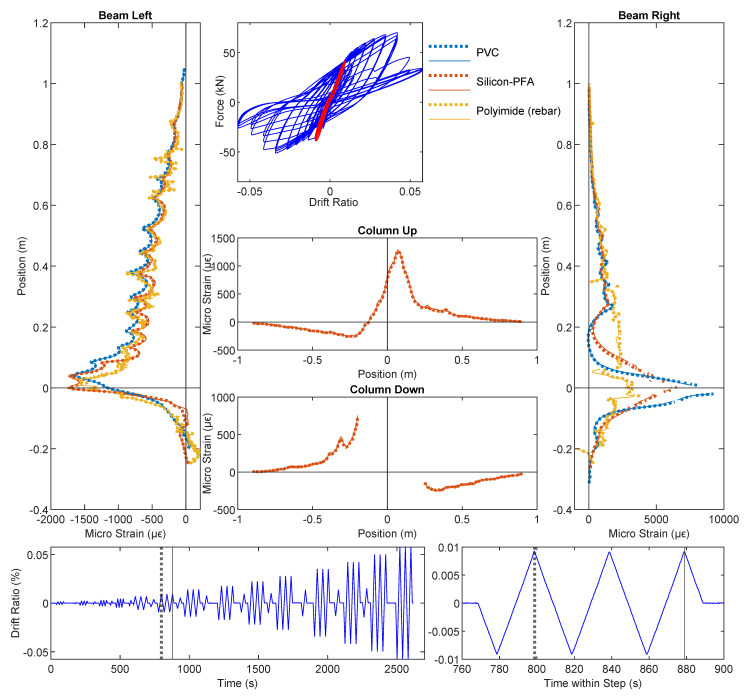
DFOS results at drift level 0.95%.

The DIC results at drift levels ±0.95% are shown in Figure 7 and the DFOS results are summarized in Figure 8 and Figure 9. A small amount of energy dissipation could already be observed at this drift level (force–drift relation in Figure 8). In Section 3.1, the DIC at ±0.14% failed to reveal any cracking information. However, at drift levels ±0.95%, the cracks along the left side of the beam were visible through DIC when the left side of the beam was under tension (negative drift, Figure 7a). The locations of these cracks confirm the likely existence of these cracks observed in DFOS at the ±0.14% drift level.

With positive drift, the cracks on the right side of the beam were not visible (Figure 7b) because when the right side of the beam underwent tension, the deformation concentrated more near the slot. This difference in the deformation mechanism can be further confirmed by the DFOS results in Figure 8 and Figure 9. While the strain gradually increased or decreased on the left side of the beam, the deformation was concentrated near the slot on the right side of the beam.

As in Section 3.1, for the drift level of ±0.95%, the two optical cables embedded in concrete (PVC and Silicon-PFA) still presented similar strain distributions compared with the optical fiber embedded in rebar on the left side of the beam. The influence of the fiber coating is now more evident: the local peaks were more prominent for the PVC cable (Figure 8), indicating the higher sensitivity of this cable. This is even more evident in the right side of the beam, where the strain distribution in the PVC cable was much larger and more concentrated (Figure 8). In this case, the extreme difference in sensitivity was likely caused by more debonding between the Silicon-PFA cable and concrete on either side of the slot.

### 3.3. Drift Level = ±1.76%

**Figure 10:**
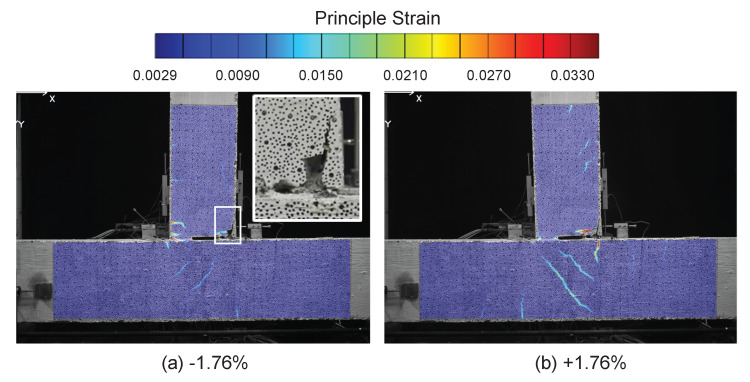
DIC results at a drift level of ±1.76%.

**Figure 11:**
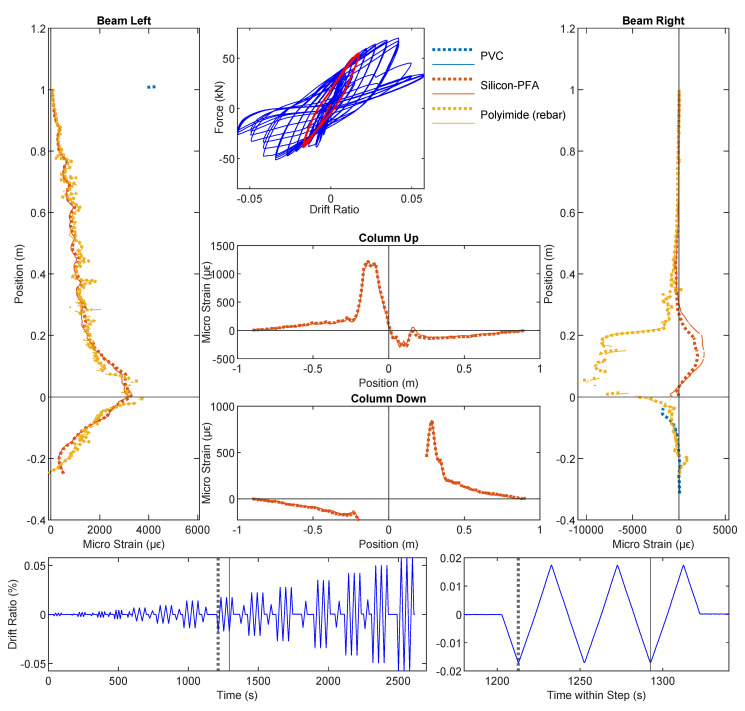
DFOS results at a drift level of −1.76%.

**Figure 12:**
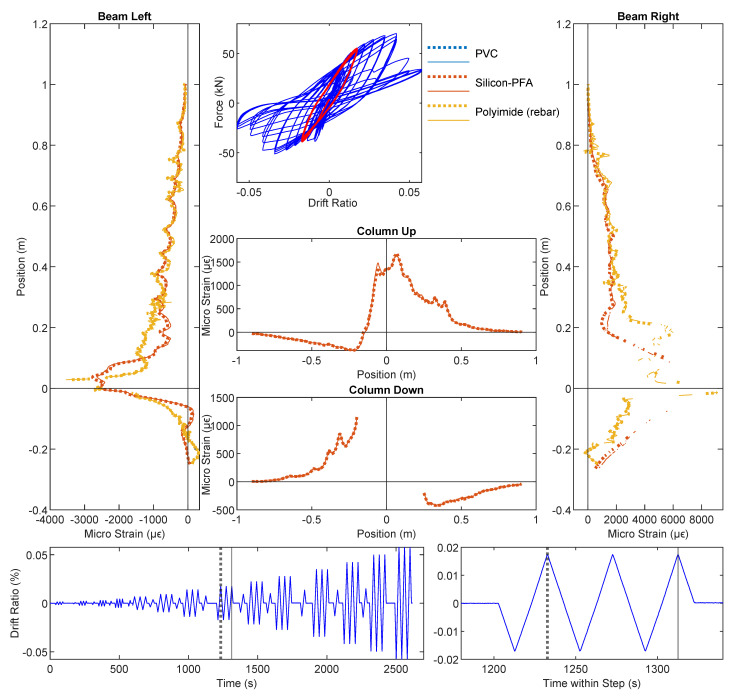
DFOS results at a drift level of 1.76%.

At a drift level of ±1.76%, DIC and DFOS results are presented in Figure 10, Figure 11 and Figure 12, respectively. Energy dissipation under cyclic loading was more prominent at this drift level (force–drift relation in Figure 11 and Figure 12). Compared with the previous drift levels, the DIC results also revealed an increasing number of cracks and larger crack widths (Figure 10), which further indicates an increasing level of damage.

Before discussing the loss of DFOS data due to cable damage, the strain distributions from the available DFOS data can be used to interpret the increased level of RC damage. In Figure 11, compressive yielding of the rebar was visible across the slot and the 180-mm unbonded length in the right side of the beam. Meanwhile, local damage to the concrete at that location (see the inset picture in Figure 10a) caused the Silicon-PFA cable to buckle. This buckling, combined with likely debonding and slippage between the Silicon-PFA cable and the concrete, as discussed in the previous section, resulted in a net positive strain at this damaged location. Meanwhile, damage was also evident in the left side of the beam, where a concentration of larger strains occurred across approximately 0.1 m of the length on the opposite side of the slot.

At a drift level of ±1.76%, the data quality deteriorated (i.e., the proportion of non-valid strain readings increased). Strain measurement using the PVC-coated cable was only possible up to the slot location in the beam. The cable must have been damaged at the slot, and no signal was obtained beyond the slot. Meanwhile, strain measurement using the Silicon-PFA-coated fiber was still possible. This was partially caused by the different directions of the cable layout (Figure 3). Starting from the fiber-optic sensing instrument (laser source), the PVC-coated cable entered the beam from the right side (enters the slot), so all data were lost beyond the damage at the slot, while the Silicon-PFA-coated cable entered from the left side and went up along the left side and down along the right side before entering the slot, after which the data were again lost (Figure 11). This observation indicates the possibility of obtaining OFDR results before the damaged part of the fiber but not after it.

Complete loss of data after the first cycle was also observed for the PVC-coated cable, which is not surprising since less debonding between the PVC cable and the concrete was observed in the previous section. The resulting large strains damaged the cable completely. Meanwhile, only a partial loss of data was observed for the Silicon-PFA cable. Some data were obtained “beyond” the slot for both the first and third cycles (Figure 12). Some debonding likely protected the cable from complete damage.

For the fiber embedded in the rebar (polyimide), a deterioration in data quality could also be observed in the form of missing (invalid) data points along the length of the fiber-optic cable but particularly across the slot for both loading directions. The high strains and strain gradients limited the ability of the analyzer to detect peaks in the frequency spectra. Note that from the LVDT results, the maximum opening of the slot was 3.20 mm for a drift level of −1.76%, which can be used to infer a strain limit for the PVC- and Silicon-PFA-coated cables. A more detailed investigation on the data quality is presented in Section 3.4. Note that similar bonding techniques between the fiber-optic cable and rebar were investigated in detail in [27] for a much more robust fiber-optic cable. The reported data quality was better, likely owing to the more significant smoothing effect from the strain-transferring mechanism of the robust cable [16].

It is worth noting that, even with a significant amount of damage at a drift level of ±1.76%, the strain for the Silicon-PFA cable was still similar to the rebar strain (polyimide) on the left side of the beam (Figure 11 and Figure 12). On the right side of the beam near the slot, the rebar yielded under compression (Figure 11), as noted earlier. Some data were lost at this location, but enough data were still obtained to interpret the behavior. Furthermore, at a drift level of ±1.76% with significant yielding at both sides of the beam, the DFOS results were still repeatable under cyclic loading for the polyimide cable in rebar and Silicon-PFA cable in concrete (Figure 11 and Figure 12). This indicates the feasibility of using these cables for cyclic loading under moderate damage.

From the DFOS results in the column, the difference in strain distributions for the two loading directions was also more evident at a drift level of ±1.76%. More specifically, negative drift (loading from right to left) caused a larger strain in the column compared with positive drift (loading from left to right). Because the reinforcement in the column was symmetric, this indicates a stronger stiffness of the beam under negative drift, which is expected due to the asymmetry of reinforcement.

### 3.4. OFDR Signal Reconstruction and Comparison with LVDT for Slot Opening

**Figure 13:**
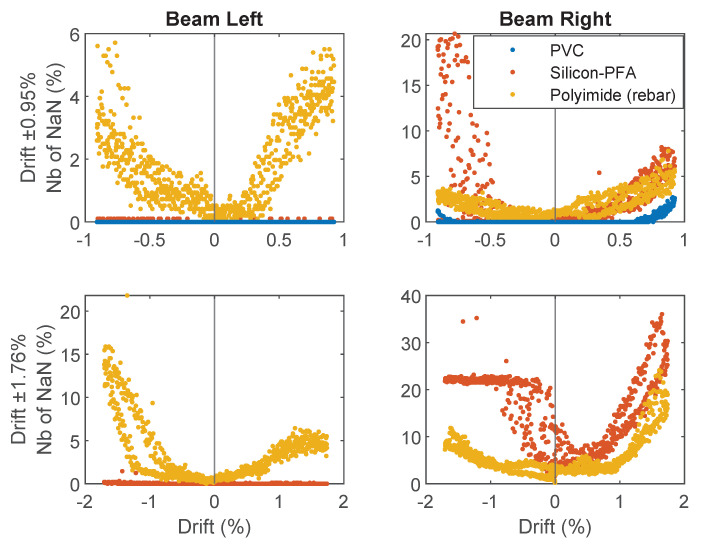
Relation between percentage of NaN and drift ratio for different fiber-optic cables under two different loading steps.

This subsection investigates the data quality of the DFOS results in more detail. Figure 13 shows the relation between the percentage of invalid points from the OFDR results and the drift level. Among the four subfigures in Figure 13, the two rows represent loading cycles with maximum drift levels of 0.95% and 1.76%, while the two columns represent the two sides of the beam.

As shown in Figure 13, the percentage of Not a Number (NaN) positively correlated with the drift level. For a maximum drift level of 0.95% on the left side of the beam (without the slot), the PVC cable and Silicon-PFA cable showed similar performances with a few invalid points. For the same drift level on the right side of the beam (with the slot), the maximum percentage of NaN for the Silicon-PFA cable reached 20% under compression (negative drift), while the percentage of NaN points for the PVC cable was still less than 3%. For a maximum drift level of 1.76%, the maximum percentage of NaN for the Silicon-PFA cable reached 35%, but notice that much of the data were still recovered when the strain was reduced again because the cable was not completely damaged. Meanwhile, the PVC cable broke at this drift level (therefore, the data are not shown in the figure).

**Figure 14:**
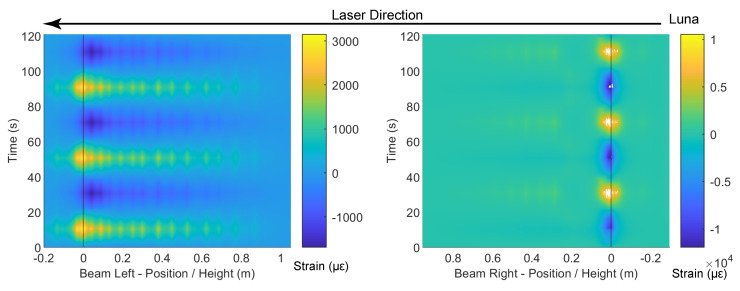
Contour of the fiber-optic strain measurements for PVC cable at a drift level of 0.95%.

**Figure 15:**
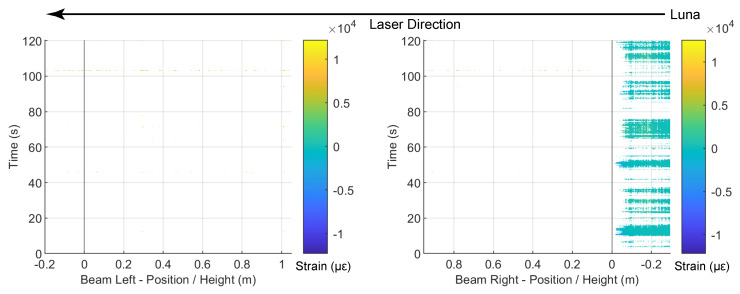
Contour of the fiber-optic strain measurements for PVC cable at a drift level of 1.76%.

**Figure 16:**
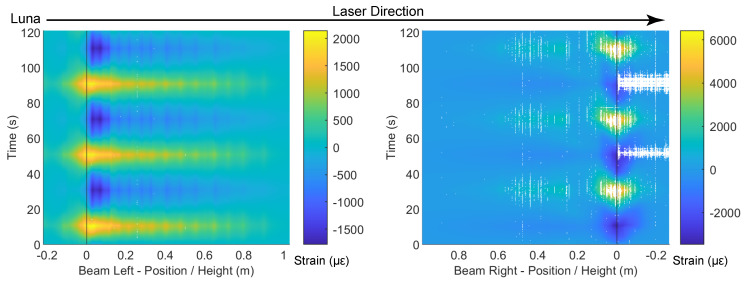
Contour of the fiber-optic strain measurements for Silicon-PFA cable at a drift level of 0.95%.

**Figure 17:**
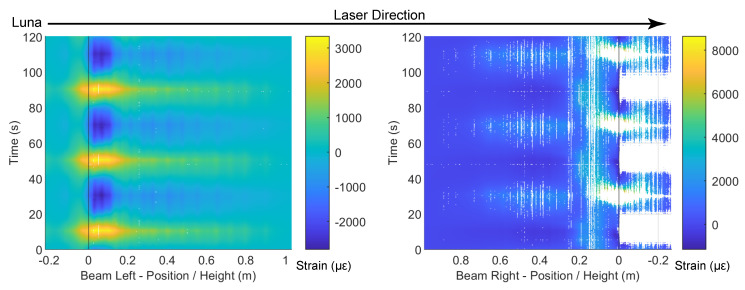
Contour of the fiber-optic strain measurements for Silicon-PFA cable at a drift level of 1.76%.

**Figure 18:**
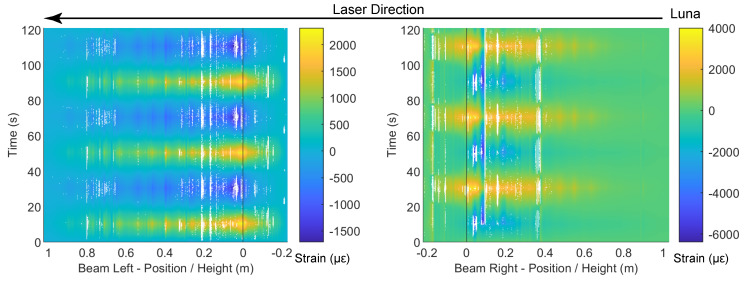
Contour of the fiber-optic strain measurements for polyimide cable (in rebar) at a drift level of 0.95%.

**Figure 19:**
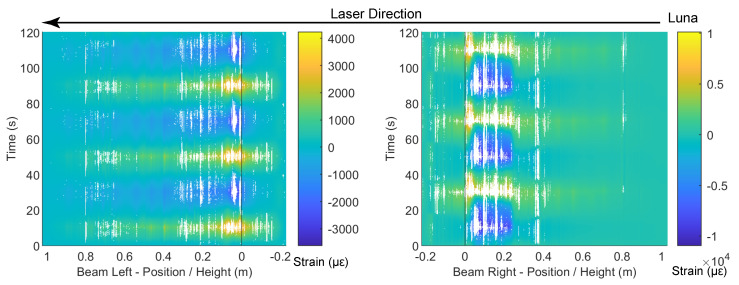
Contour of the fiber-optic strain measurements for polyimide cable (in rebar) at a drift level of 1.76%.

To investigate the location of the invalid points and the influence of the laser direction, the DFOS data are presented in contour plots in Figure 14, Figure 15, Figure 16, Figure 17, Figure 18 and Figure 19. Figure 14 and Figure 15 present the DFOS data for the PVC cable under cyclic loading when the maximum drift was 0.95% and 1.76%, respectively. Note that the direction of the *x*-axis in the figure is set so that the laser direction is continuous (i.e., the laser enters the specimen from the bottom right side of the beam and exits from the bottom left side (Figure 3)). From Figure 14, three loading cycles can be clearly identified at a drift level of 0.95%. When the drift level increases from 0.95% to 1.76%, because of the cable breakage near the slot, most of the data become invalid. Only the data from the initial portion of the cable (before reaching the breakage point) are partially available.

Similarly, Figure 16 and Figure 17 present the DFOS data for the Silicon-PFA cable, for which the cable direction is reversed (Figure 3). As observed in Figure 13, a comparison of Figure 14 and Figure 16 indicates that the PVC cable showed better data quality when the maximum drift level was 0.95%. At a drift level of 1.76%, because of the cable direction, the data were mostly recorded for the Silicon-PFA cable before reaching the slot. It is also worth noting that, unlike the PVC cable, the Silicon-PFA cable was not completely broken at the slot; it was still able to sense beyond the slot during the unloading process. Finally, Figure 18 and Figure 19 present DFOS data for the polyimide cable in the rebar. Note that the cable direction (*x*-axis) was, again, different (Figure 3). In this case, the cable remained undamaged, so the laser direction was not important.

**Figure 20:**
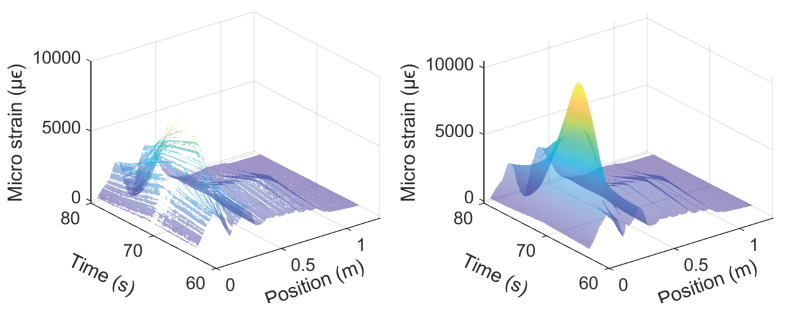
Reconstruction of the fiber-optic strain measurement (Silicon-PFA) for the right side of the beam at a drift level of 1.76%.

**Figure 21:**
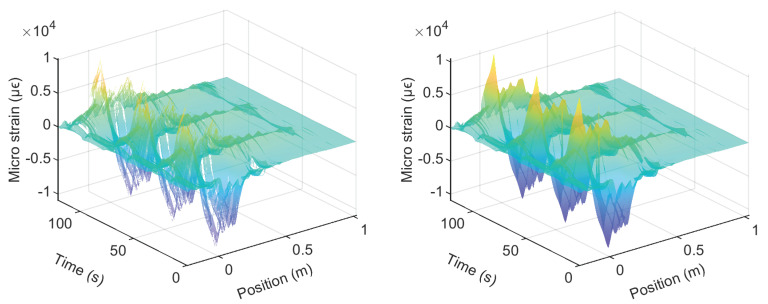
Reconstruction of the fiber-optic strain measurement (Polyimide, rebar) for the right side of the beam at a drift level of 1.76 %.

To mitigate the loss of data observed above, interpolation can be used to fill in missing data. A 2D interpolation algorithm that uses a plate equation (realized in Matlab using inpaint_nans) was adopted here to achieve simultaneous interpolation both in the temporary and spatial domains. At a drift level of 1.76%, the strain before and after 2D interpolation to fill in the missing data are shown in Figure 20 for the Silicon-PFA cable and in Figure 21 for the polyimide cable in the rebar. Note that the interpolation for the Silicon-PFA cable was conducted at selected intervals because of the presence of large regions of missing data (Figure 17). The visual comparison shows satisfactory results for the adopted interpolation algorithm.

**Figure 22:**
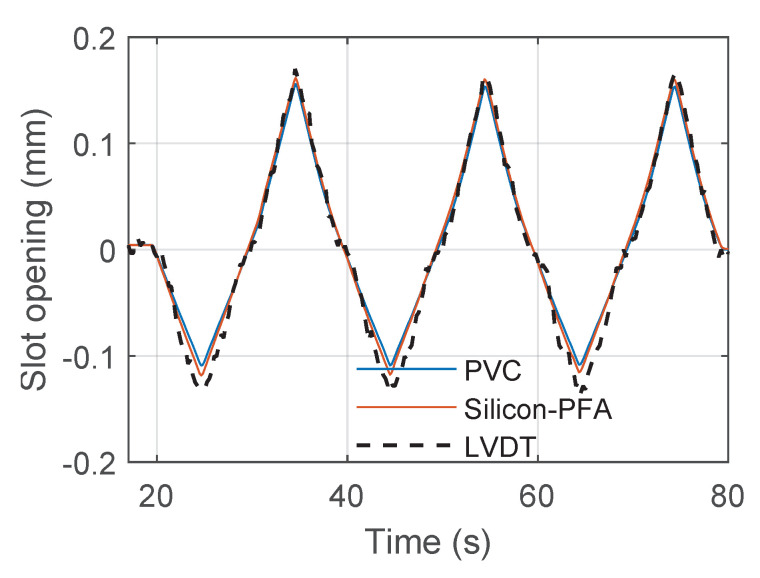
Comparison of fiber-optic and LVDT measurements of slot opening at a drift level of 0.14%.

**Figure 23:**
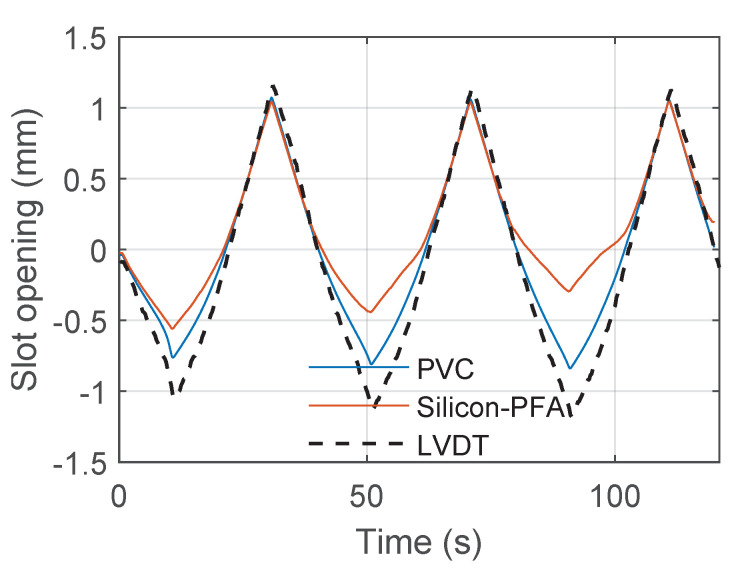
Comparison of fiber-optic and LVDT measurements of slot opening at a drift level of 0.95%.

**Figure 24:**
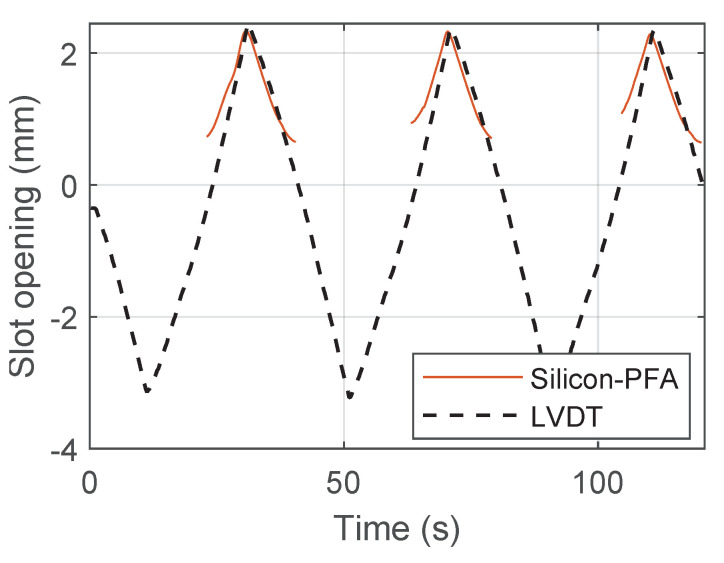
Comparison of fiber-optic and LVDT measurements of slot opening at a drift level of 1.76%.

After signal reconstruction, it was possible to integrate the strain in the fiber-optic cable near the slot to obtain the slot opening and closing. Figure 22, Figure 23 and Figure 24 present the comparison between the slot opening and closing obtained from integrating DFOS strain and from LVDT measurements (Figure 2a). For a drift of 0.14% (Figure 22), the accuracy of DFOS in measuring the slot opening and closing was relatively high. For a drift of 0.95%, DFOS was accurate in measuring the slot opening, but the error started to increase during slot closing (Figure 23). This was unique to this beam–column connection type, for which the slot and debonded length caused the cable to buckle in compression because of concrete damage (see Section 3.2). For a drift of 1.76%, it was only possible to find the slot opening and closing in limited regions because of a large amount of missing data.

## 4. Damage Quantification

The previous sections presented the DFOS results at selected drift levels and focused on explaining the mechanical behavior and quality of the DFOS results. This section investigates the feasibility of using DFOS to quantify structural damage, which would be useful for post-earthquake structural evaluation. As a first study, several indices are proposed based on the DFOS results, and their correlations with the maximum sustained drift of the specimen are investigated. The maximum sustained drift is a typical indicator of damage severity [28].

While an innovative RC joint was tested in this paper, to make the discussion more representative for common RC structures, only the information on the left side of the beam (without a slot in Figure 3) was used. Because the Silicon-PFA cable had a higher survivability, which allowed investigation at larger drift levels, as a first investigation, the damage indices were derived from the Silicon-PFA cable in this section.

**Figure 25:**
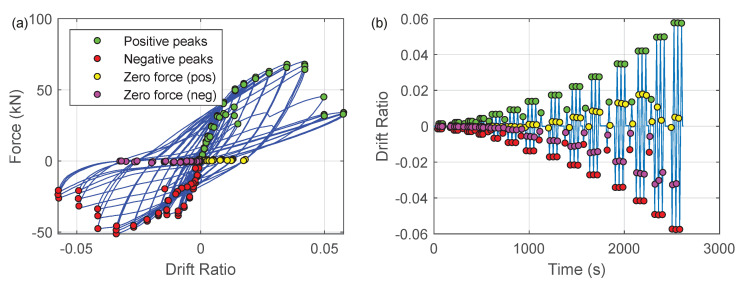
(**a**) Force–drift ratio relationship. (**b**) Drift ratio history.

For each loading cycle, four different characteristic loading instants were investigated (as shown in the force–drift relation in Figure 25):

- Positive peaks: the loading points corresponding to the maximum drift at each loading cycle (note that positive drift is defined as loading from right to left in Figure 3);
- Negative peaks: the loading instants corresponding to the minimum drift at each loading cycle;
- Zero force (pos): the loading points when the force first returns to zero after reaching “positive peaks”. Note that this is essentially the residual drift after unloading from a positive peak;
- Zero force (neg): the loading points when the force first returns to zero after reaching “negative peaks”. Note that this is essentially the residual drift after unloading from a negative peak.

**Figure 26:**
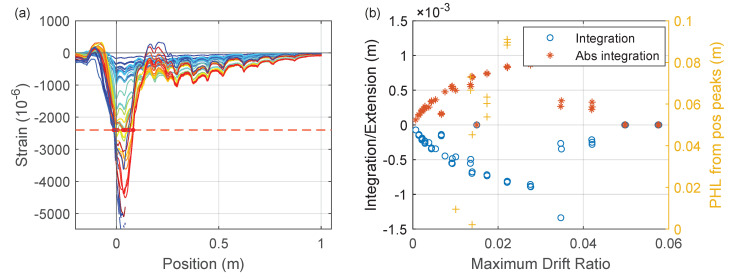
(**a**) Strain measurements at positive peaks of Beam Left (Silicon-PFA). (**b**) Proposed damage indices from maximum DFOS strain readings vs. maximum drift.

**Figure 27:**
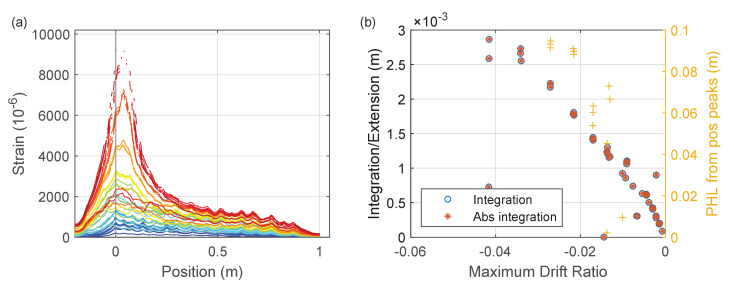
(**a**) Strain measurements at negative peaks of Beam Left (Silicon-PFA). (**b**) Proposed damage indices from maximum DFOS strain readings vs. maximum drift.

Because the maximum drift was achieved at the positive and negative peaks (Figure 26a and Figure 27a), conceptually, it is more desirable to use the DFOS results at the positive and negative peaks to evaluate the maximum drift. Provided that the strain information is available from DFOS along both sides of the beam, the curvature can be estimated from the two opposing strain values along the length of the beam, with which the maximum drift can be estimated through integration of the curvature [13].

**Figure 28:**
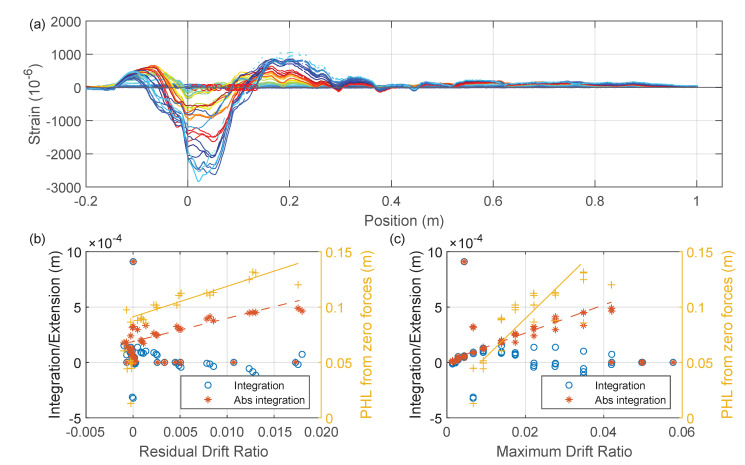
(**a**) Strain measurements at zero forces with positive drifts for A2-Beam left (down to up Silicon-PFA). (**b**) Proposed damage indices from residual DFOS strain readings vs. residual drift. (**c**) Proposed damage indices from residual DFOS strain readings vs. maximum drift.

**Figure 29:**
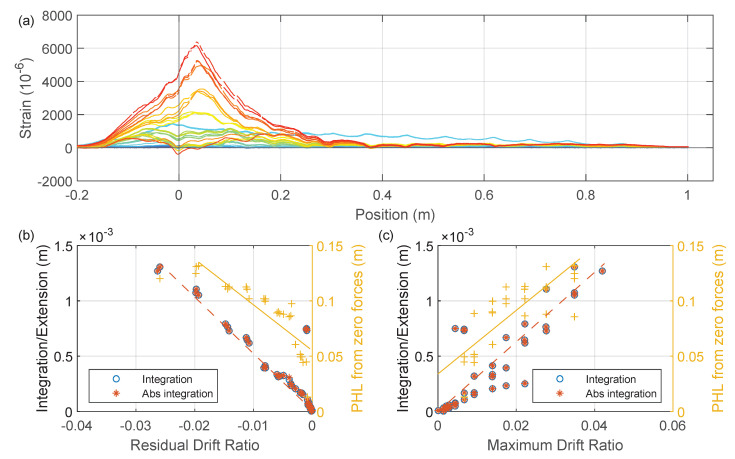
(**a**) Strain measurements at zero forces with negative drifts for A2-Beam left (down to up Silicon-PFA). (**b**) Proposed damage indices from residual DFOS strain readings vs. residual drift. (**c**) Proposed damage indices from residual DFOS strain readings vs. maximum drift.

However, the DFOS results at the peaks are not often available, especially for on-site applications. This is because (1) the measurement frequency of the DFOS technology adopted might not be high enough for real time evaluation (e.g., Brillouin-based sensing technology usually takes several minutes to finish one reading), (2) the DFOS analyzer might not be available or in operation during the earthquake, and measurements are more likely to be taken after the earthquake event. In these cases, only the residual DFOS results would be available (when the force returns to zero; Figure 28a and Figure 29a). Therefore, it would be beneficial to use the residual DFOS results to evaluate the damage (i.e., to predict the maximum drift). For post-earthquake structural evaluation, residual drift is also an important indicator [29]. For these reasons, the correlation between the proposed indices and residual drift will also be investigated.

The DFOS results at the positive peaks are summarized in Figure 26a. The correlations of different indices with the maximum drift are presented in Figure 26b. The three indices are defined as follows:

- Integration (left *y*-axis in Figure 26b and Figure 27b): integration of DFOS strain along the length of the fiber (on the left side of the beam), which has the physical meaning of the total elongation or shortening of the fiber-optic cable.
- Abs integration (left *y*-axis in Figure 26b and Figure 27b): integrating the absolute strain from DFOS, which has the physical meaning of the “total” deformation of the fiber-optic cable.
- Plastic hinge length (PHL) from pos peaks (right *y*-axis in Figure 26b and Figure 27b): estimated from the compressive strain in the concrete core, as proposed in [30]. Note that although the PHL used here is different from the plastic hinge length commonly used in structural engineering [31], this parameter provides an estimation of the region in which longitudinal bars are yielding in compression [30].

Similar to Figure 26, Figure 27 shows the correlation between three proposed indices (integration, abs integration, and plastic hinge length (PHL)) with the maximum drift caused by the negative peaks.

Figure 28a presents the residual strain distribution when the force returns to zero after a positive peak, while Figure 28b,c provide the correlation between the defined indices (calculated from the residual DFOS strain when the force is zero) and the residual or maximum drift. Note that while “integration” and “abs integration” have the same definition as before, “PHL from zero force” is defined as the length between the beam end and first zero crossing point of the strain profile (distance between circles in Figure 28a).

As shown in Figure 28b, there was a positive correlation between “abs integration” and the residual drift. Figure 28c shows that “abs integration” also positively correlates with the maximum drift. Similarly, “PHL from zero forces” also provides a positive correlation with both residual drift (Figure 28b) and maximum drift (Figure 28c). However, in Figure 28c, “PHL from zero forces” only starts after the maximum drift reaches around 1%, which indicates that the plastic hinge did not form at the initial linear phase when the maximum drift is small. From strain measurements at zero forces with positive drift, the correlation between “integration” and maximum/residual drift is not clear.

Similarly, Figure 29 provides results which when the force returned to zero after the negative peaks. Interestingly, the strain distributions are significantly different from those in Figure 28. This was caused by the asymmetric reinforcement at the top and bottom of the beam. Negative drift creates a larger plastic deformation (Figure 27a vs. Figure 26a), which causes a more prominent residual deformation when the force returns to zero. It is worth noting that the residual strain from negative drifts has no zero crossings, and thus the PHLs from zero forces with positive drifts (Figure 28) were used for comparison. For strain measurements at zero forces with negative drift (where there is almost no zero crossing in the residual strain distribution), the two damage indices, “integration” and “abs integration”, are almost indistinguishable and both correlates well with maximum/residual drift.

To summarize, proper selection of damage indices based on DFOS strain readings after seismic events can provide valuable information about the damage level of a reinforced concrete structure, including the residual drift after an earthquake, the maximum drift experienced during the earthquake, the plastic hinge length, etc.

## 5. Conclusions and Outlook

The aim of the present study was to evaluate the capability of DFOS in sensing the deformation and damage of an RC beam–column joint subjected to cyclic loading. The conclusions are summarized as follows:

- DFOS provided detailed information about the structural behavior of the beam–column joint with much higher accuracy than DIC. For the fiber-optic cables evaluated, the strain results were consistent for cyclic loading that induced moderate damage. From the DFOS results, the asymmetry of the strain distributions in the rebar and concrete on two sides of the beam at different drift levels was clearly evident. With DFOS, the effects of the 180-mm debonded length of rebar near the slot were clearly observed, as well as the development of yielding in the reinforcement. DFOS also yielded accurate slot opening measurements until significant damage occurred in the concrete, leaving the cable loose and unbonded and therefore unable to accurately measure compressive strains across the open slot due to buckling of the fiber-optic cable.
- The influence of the cable coating and cable position were also discussed. Both PVC and Silicon-PFA (same positions in the concrete but with opposite directions) were damaged due to high strains at the slot before a drift level of −1.76% was achieved. Silicon-PFA revealed more information about the strain distribution thanks to the layout direction of the cable, which indicates the importance of the DFOS design, as well as the coating (cable jacket) material that caused the cable to partially debond from the concrete before being completely damaged. The polyimide cable (embedded in rebar) successfully recorded data for larger drift levels. It started to lose a significant amount of data at a drift level of −3.5%, which corresponds to a maximum slot opening of 6.15 mm. With moderate damage (e.g., 1.76%), the difference between the strain measured in the concrete and the rebar was small on the left side of the beam (without the slot), which is representative of typical RC members. With the appropriate cable coating, this suggests the possibility of sensing rebar deformation without embedding optical cable in the rebar, at least until high levels of damage are reached.
- Three different damage indices based on DFOS were proposed and evaluated: integration of strain (i.e., total elongation), integration of the absolute strain (i.e., total deformation), and an approximation of the plastic hinge length (PHL). These indices were evaluated both at the maximum and residual displacements. The integration of the absolute strain at the residual displacement correlated well with the maximum drift sustained by the beam–column joint. Though preliminary, this suggests the possibility of evaluating the maximum sustained damage using the residual strain obtained from DFOS measurements after an earthquake.

## Data Availability

The test data set is available on Dryad, https://doi.org/10.6078/D17H91.
